# Serological investigation of *Leishmania infantum*, *Dirofilaria immitis* and *Angiostrongylus vasorum* in dogs from southern Portugal

**DOI:** 10.1186/s13071-015-0771-z

**Published:** 2015-03-23

**Authors:** Carla Maia, Mónica Coimbra, Cláudia Ramos, José Manuel Cristóvão, Luís Cardoso, Lenea Campino

**Affiliations:** Unidade de Parasitologia Médica, Instituto de Higiene e Medicina Tropical (IHMT), Universidade Nova de Lisboa (UNL), Rua da Junqueira 100, 1349-008 Lisboa, Portugal; Global Health and Tropical Medicine, IHMT-UNL, Rua da Junqueira 100, 1349-008 Lisboa, Portugal; Faculdade de Medicina Veterinária, Universidade Lusófona de Humanidades e Tecnologias (ULHT), Lisboa, Portugal; Clínica Veterinária Porto Seguro, Rua Patrão Joaquim Casaca, Edifício Majuca, 8700-507 Olhão, Portugal; Departamento de Ciências Veterinárias, Escola de Ciências Agrárias e Veterinárias, Universidade de Trás-os-Montes e Alto Douro (UTAD), Quinta de Prados, 5000-801 Vila Real, Portugal; Departamento de Ciências Biomédicas e Medicina, Universidade do Algarve, Campus de Gambelas, 8005-139 Faro, Portugal

**Keywords:** *Leishmania infantum*, *Dirofilaria immitis*, *Angiostrongylus vasorum*, Dog, Portugal, Seroprevalence

## Abstract

**Background:**

Leishmaniosis, dirofilariosis and angiostrongylosis are parasitic diseases of established importance in dogs worldwide. The aim of the present report was to determine all together levels of infection with or exposure to *Leishmania infantum*, *Dirofilaria immitis* and *Angiostrongylus vasorum* in dogs from the Algarve region, in southern Portugal.

**Findings:**

Serum samples were obtained from a total of 170 apparently healthy dogs. Antibodies to *L. infantum* were detected by the direct agglutination test; and antigens of *D. immitis* and of *A. vasorum* by an enzyme-linked immunosorbent assay and an immunochromatography test, respectively. Antibodies to *L. infantum* were found in 18.2% (31/170) of dogs, while *D. immitis* antigen was detected in 9.4% (16/170). None of 120 dogs tested out of the 170 under investigation had evidence of infection with *A. vasorum. D. immitis* infection was more prevalent in dogs aged 84–204 months than in those younger than 12 months. Three dogs were positive to both *L. infantum* and *D. immitis*.

**Conclusions:**

Dogs living in southern Portugal are at risk of leishmaniosis and dirofilariosis. This scenario should make the veterinary community, local dog owners and also tourists from non-endemic countries coming on vacation with their pets aware of the need of effective prophylactic measures, in order to protect animals and public health.

## Findings

Leishmaniosis, dirofilariosis and angiostrongylosis are parasitic diseases of established importance in dogs worldwide. Leishmaniosis caused by the protozoan *Leishmania infantum* (Trypanosomatida, Trypanosomatidae) and heartworm disease caused by the nematode *Dirofilaria immitis* (Spirurida, Onchocercidae) are vector-borne zoonoses with importance in veterinary medicine and widely present in the Mediterranean [1-3]. Both parasitoses are endemic in Portugal, with an overall national seroprevalence of 6.3% [4] and 3.6-8.9% [5], respectively.

Canine leishmaniosis is a systemic chronic condition whose clinical manifestations usually include lymphadenomegaly, cutaneous alterations, loss of body weight, ocular signs, epistaxis, onychogryphosis and lameness [6]. Canine cardiopulmonary dirofilariosis is associated with a dry chronic cough, exercise intolerance, dyspnoea, weakness, weight loss, epistaxis, cyanosis and even congestive heart failure [7].

Canine angiostrongylosis, caused by the nematode *Angiostrongylus vasorum* (Strongylida, Angiostrongylidae), is an emerging disease with an increasing number of cases diagnosed in the last few years, and with a high established relevance in several regions of Europe [8]. The disease is associated with an array of clinical problems, including cardiorespiratory, coagulopathic and neurologic signs [8]. Albeit the laboratory Baermann method, using faeces, is the gold-standard to detect first-stage larvae (L1), serological techniques for the detection of circulating *A. vasorum* antigens or specific antibodies have recently been developed [9,10]. In Europe prevalence as assessed by the detection of circulating parasite antigens in dogs has been found to range from 0.5% in Germany to 1.3% in the United Kingdom, while the detection of specific antibodies ranged from 1.3% in Poland to 3.2% in the UK [11,12]. In coastal areas of south-central Portugal, 1.8% (6/341) of shelter dogs were antigen-positive, while antibodies against the parasite were detected in 2.4% of those dogs [13].

The present report aimed at determining the levels of infection with or exposure to vector-borne parasites (*L. infantum* and *D. immitis*) and parasites acquired by ingestion of intermediate hosts (*A. vasorum*) in dogs from the Algarve region, the southernmost part of continental Portugal, in view of the scarcity of epidemiological studies targeting these parasites all together in canine populations.

From November 2011 to May 2014, a total of 170 apparently healthy dogs from the Algarve were studied. Domestic dogs were randomly included after owners’ informed consent. In the case of stray dogs, a written consent for enrolment was obtained from the legal detainer, i.e. the person in charge of the rescue association. Whenever available, data on gender, breed, age, fur length, life style, living conditions, prophylactic use of sand fly repellents and of macrocyclic lactones were registered for each dog (Tables 1 and 2). Blood samples (2–3 ml) were collected by cephalic or jugular venipuncture. Serum was separated by centrifugation and stored at −20°C until use.

**Table 1:** **Prevalence of antibodies to** ***Leishmania infantum*** **in dogs from southern Portugal according to independent variables and their categories**

**Independent variable/category**	**No. (%) of dogs tested**	**% of positive**	**95% CI**
Gender	169	*p* = 0.161	
Female	79 (46.7)	22.8	7.1–22.1
Male	90 (53.3)	13.3	14.1–33.6
Breed	167	*p* = 0.747	
Defined	93 (55.7)	19.4	8.7–26.6
Mongrel	74 (44.3)	16.2	11.9–28.8
Fur length	81	*p* = 0.393	
Short	47 (58.0)	10.6	3.5–23.1
Medium or long	34 (42.0)	2.9	0.1–15.3
Age (months)	155	*p* = 0.530	
[2–11]	31 (20.0)	16.1	5.5–33.7
[12–83]	74 (47.7)	21.6	12.9–32.7
[84–204]	50 (32.3)	14.0	5.8–26.7
Lifestyle	170	*p* = 1.0	
Domestic	157 (92.4)	18.5	12.7–25.4
Stray	13 (7.6)	15.4	1.9–45.4
Housing	161	*p* = 0.280	
Exclusively or mainly indoors	69 (42.9)	23.2	13.9–34.9
Mainly or exclusively outdoors	92 (57.1)	15.2	8.6–24.2
Sand fly repellents*	145	*p* = 1.0	
Yes	58 (46.9)	17.6	9.5–28.8
No	77 (53.1)	16.9	9.3–27.1
*D. immitis*	170	*p* = 1.0	
Negative	154 (90.6)	18.2	12.4–25.2
Positive	16 (9.4)	18.8	4.0–45.6
Total	170	18.2	12.7–24.7

*Deltamethrin and/or imidacloprid and permethrin.

**Table 2:** **Prevalence of** ***Dirofilaria immitis*** **antigen in dogs from southern Portugal according to independent variables and their categories**

**Independent variable/category**	**No. (%) of dogs tested**	**% of positive**	**95% CI**
Gender	169	*p* = 0.297	
Female	79 (46.7)	6.3	2.1–14.2
Male	90 (53.3)	12.2	6.3–20.8
Breed	167	*p* = 0.071	
Defined	93 (55.7)	5.4	1.8–12.1
Mongrel	74 (44.3)	14.9	7.7–25.0
Fur length	81	*p* = 1.0	
Short	47 (58.0)	6.4	1.3–17.5
Medium or long	34 (42.0)	5.9	0.7–19.7
Age (months)	155	*p* = ND	
[2–11]	31 (20.0)	0.0^a^	0.0–11.2
[12–83]	74 (47.7)	9.5	3.9–18.5
[84–204]	50 (32.3)	16.0^a^	7.2–29.1
Lifestyle	170	*p* = 0.352	
Domestic	157 (92.4)	8.9	5.0–14.5
Stray	13 (7.6)	15.4	1.9–45.4
Housing	161	*p* = 1.0	
Exclusively or mainly indoors	69 (42.9)	8.7	3.3–18.0
Mainly or exclusively outdoors	92 (57.1)	9.8	4.6–17.8
Macrocyclic lactones*	143	*p* = 0.128	
Yes	23 (16.1)	0.0	0.0–14.8
No	120 (83.9)	10.8	5.9–17.8
*Leishmania* spp.	170	*p* = 1.0	
Negative	139 (81.8)	9.4	5.1–15.4
Positive	31 (18.2)	9.7	2.0–25.7
Total	170	9.4	5.5–14.8

*Ivermectin and/or milbemycin oxime or moxydectin; ^a^ *p* = 0.021.

This study was approved by the ethics board of the Institute of Hygiene and Tropical Medicine, Universidade Nova de Lisboa (IHMT-UNL) as complying with the Portuguese legislation for the protection of animals (Law no. 92/1995).

The direct agglutination test (DAT) for titration of specific antibodies was performed in 170 dogs using a standard freeze-dried *Leishmania* antigen as previously described [14]. The DAT cut-off was established at a titre of 400 [15]. *D. immitis* antigen was detected in 170 animals by using a commercial enzyme-linked immunosorbent assay kit (PetChek Canine Heartworm Antigen Test®, IDEXX Laboratories, Westbrook, ME, USA). Detection of *A. vasorum* antigens was performed in only 120 dogs out of the 170 under investigation by using a commercial immunochromatography test (Angio Detect™ Test, IDEXX Laboratories).

The exact binomial test established confidence intervals (CI) with a 95% confidence level. The chi-square or Fisher’s exact tests were used to compare percentages of positivity among categories of the same independent variables and also the total prevalence of each agent [16]. A *p* value < 0.05 was considered as statistically significant. Analyses were performed with StatLib and SPSS® 21 software for Windows.

Antibodies to *L. infantum* were found in 31 (18.2%) dogs, at the dilutions of 1:400 (n = 5), 1:800 (n = 2), 1:1,600 (n = 2), 1:3,200 (n = 5), 1:6,400 (n = 6), 1:12,800 (n = 6), 1:25,600 (n = 4) and 1:51,200 (n = 1) (Table 1). The seroprevalence of *Leishmania* found in the present study was higher than the ones obtained in randomly screened animals (4.7%) [4] and in clinically healthy dogs (3.8%) [5] from the Algarve region that were presented to veterinary clinics, suggesting an apparent increase in the level of *Leishmania* infection in the Algarve [17].

*D. immitis* antigen was detected in 16 (9.6%) animals (Table 2). This prevalence is apparently higher than the values recently obtained in three coastal areas of south-central Portugal (7.9%) [13] and in other areas further north (2.1%) [18], suggesting that the climatic conditions in the southern regions of the country might be more favourable to the proliferation and abundance of vectors of *D. immitis* [19,20]. An exception to this trend was reported in Figueira da Foz, located in the coastal edge of the central region of Portugal, where 25.7% of the dogs were found infected [21].

Prevalence of *D. immitis* infection was significantly higher in dogs aged 84–204 months than in dogs younger than 12 months (*p* = 0.021), which may be explained by a cumulative exposure of older animals to the parasite through an increased contact with the vectors [5,21]. Although the difference was not statistically significant (*p* = 0.128), *D. immitis* antigens were not detected in any of the animals receiving macrocyclic lactones; anyhow, this finding suggests the importance of using prophylaxis in areas where dirofilariosis is endemic.

Cross-reactivity between *D. immitis* and *A. vasorum* in dogs experimentally infected with the latter has previously been reported for some heartworm antigen test kits [22]. However, in the present study no cross-reaction between both nematodes seem to have happened, as *A. vasorum* antigens were not detected in any of the dogs tested (0.0%; 95% CI: 0.0-3.0). The non-detection of this parasite, in comparison with the survey (1.8%) carried out in other coastal areas of Portugal [14], might be related with the sensitivity of the present test (i.e. 98.1% [23]), with seasonal factors and/or with the surveyed canine populations, as in the previous work all animals were shelter dogs, probably at a higher risk of contact with the intermediate host (i.e. snails and slugs), while in the present study most of the dogs were pets. Nevertheless, larger surveys and using different techniques (Baerman method, detection of specific antibodies and antigens) are necessary to gain more information on the diffusion of *A. vasorum*, for which the actual national distribution is poorly acquainted.

In the present study, no statistical association was found between positivities to *L. infantum* and *D. immitis*. Only three dogs were positive to both *L. infantum* and *D. immitis*. Co-infections with these two vector-borne pathogens are relatively frequent in dogs living in geographic areas where the presence of competent vectors for the different pathogens overlap [5,24]. Although in this study the three animals positive to both parasites were apparently healthy, it is important to keep in mind that the occurrence of co-infections with vector-borne pathogens might induce uncharacteristic clinical outcomes that will further complicate diagnosis, treatment and prognosis [25].

The findings of this study reveal that dogs from southern Portugal are at risk of acquiring two of the three parasites assessed (i.e. the agents of leishmaniosis and dirofilariosis). They also reinforce the importance to alert the veterinary community, local dog owners as well as tourists from non-endemic countries coming on vacation with their pets to the need of prophylactic measures, such as insect repellents and macrocyclic lactones, in order to defend animals and public health.
